# First Whole-Genome Sequencing Analysis of Tracheobronchopathia Osteochondroplastica with Critical Vocal Cord Involvement: Proposing a Novel Pathophysiological Model

**DOI:** 10.3390/diagnostics16020210

**Published:** 2026-01-09

**Authors:** Yeonhee Park, Joo-Eun Lee, Mi Jung Lim, Hyeong Seok Kang, Chaeuk Chung

**Affiliations:** 1 Division of Pulmonary and Critical Care Medicine, Department of Internal Medicine, Daejeon St. Mary’s Hospital, College of Medicine, The Catholic University of Korea, Seoul 06591, Republic of Korea; yhpark@catholic.ac.kr; 2 Division of Pulmonology and Critical Care Medicine, Department of Internal Medicine, College of Medicine, Chungnam National University, Daejeon 35015, Republic of Korea; jooeunlee83@gmail.com (J.-E.L.); wooayyak@daum.net (H.S.K.); 3 Genomics Department, Keyomics Co., Ltd., Yuseong-gu, Daejeon 34013, Republic of Korea; mjlim@keyomics.co.kr

**Keywords:** tracheobronchopathia osteochondroplastica, whole genome sequencing, acute respiratory failure

## Abstract

**Background**: Tracheobronchopathia osteochondroplastica (TO) is a rare benign disorder characterized by submucosal cartilaginous and osseous nodules of the tracheobronchial tree, typically sparing the posterior membranous wall. Involvement of the vocal cords is exceedingly rare and may result in critical airway obstruction. The underlying genetic and molecular mechanisms of TO remain largely unexplored. **Case presentation**: We report a rare case of TO extending from the vocal cords to the bronchi in a 76-year-old man who initially presented with pneumonia and later developed acute respiratory failure due to severe airway narrowing, necessitating emergency tracheostomy. Bronchoscopy and computed tomography revealed diffuse calcified nodules involving the anterior and lateral airway walls, including the subglottic region. Histopathology demonstrated chronic inflammatory cell infiltration with squamous metaplasia. To explore the molecular basis of this condition, whole-genome sequencing (WGS) was performed using peripheral blood samples—the first such application in TO. WGS identified 766 germline mutations (including 27 high-impact variants) and 66 structural variations. Candidate genes were implicated in coagulation and inflammation (*KNG1*), arachidonic acid metabolism and extracellular matrix remodeling (*PLA2G4D*), ciliary dysfunction and mineralization (*TMEM67*), vascular calcification (*CDKN2B-AS1*), smooth muscle function (*MYLK4*), abnormal calcification (*TRPV2, SPRY2, BAZ1B*), fibrotic signaling (*AHNAK2*), and mucosal barrier integrity (*MUC12/MUC19*). Notably, despite systemic germline mutations, calcification was restricted to the airway. **Conclusions**: This case highlights that TO with vocal cord involvement can progress beyond a benign course to cause life-threatening airway obstruction. Integrating clinical, histological, and genomic findings, we propose a novel pathophysiological model in which systemic genetic susceptibility interacts with local immune cell infiltration and fibroblast-driven extracellular matrix remodeling, resulting in airway-restricted dystrophic calcification. This first genomic characterization of TO provides new insights into its pathogenesis and suggests that multi-omics approaches may enable future precision medicine strategies for this rare airway disease.

## 1. Introduction

Tracheobronchopathia osteochondroplastica (TO) is a rare, benign disorder of the tracheobronchial system. It is marked by submucosal cartilaginous and osseous nodules that protrude into the airway lumen, as seen during bronchoscopy. This condition creates a unique “cobblestone” appearance, while the posterior tracheal wall remains unaffected. The exact prevalence remains uncertain, with estimates in autopsy and bronchoscopy series ranging from 0.01% to 0.3% [1,2].

TO typically exhibits a gradual progression and is frequently identified incidentally. When symptoms manifest, they may include chronic cough, dyspnea, recurrent infections, or wheezing [3,4]. In most patients, the clinical course is benign; however, in rare cases, extensive airway involvement may cause significant morbidity, including severe airway obstruction and acute respiratory failure [5,6]. Instances of TO extending to the vocal cords are extremely rare and present additional risks of airway compromise.

Although the pathology of TO is distinct, its etiology remains unclear. The proposed hypotheses include chronic inflammation, environmental exposure, mechanical irritation, and metabolic abnormalities; however, none has been definitively established [7]. To our knowledge, no previous studies have investigated the genomic or molecular basis of TO. Here, we present a rare case of TO involving the vocal cords, complicated by pneumonia and subsequent acute airway obstruction requiring tracheostomy. We further report the first application of whole-genome sequencing (WGS) in TO, providing new insights into its potential genetic underpinnings.

## 2. Case Presentation

**Figure 1:**
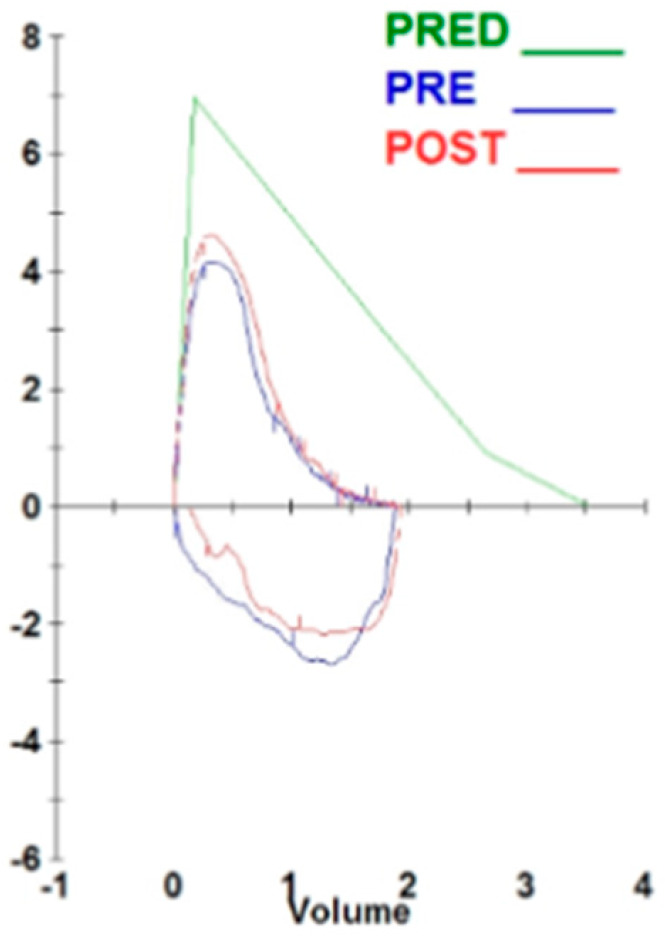
The flow–volume loop obtained from pulmonary function testing. The flow–volume loop reveals a diminished expiratory flow with a concave shape, indicative of obstructive ventilatory impairment. The curves for predicted (PRED, green line), pre-bronchodilator (PRE, blue line), and post-bronchodilator (POST, red line) are displayed. There was no notable bronchodilator reversibility detected.

The patient was a 76-year-old male ex-smoker (10 pack-years, quit 40 years ago) and had no history of pulmonary tuberculosis. He had been previously diagnosed with COPD at a local clinic, reported chronic dyspnea with an mMRC grade of 2–3. Upon initial assessment, pulmonary function tests revealed a forced expiratory volume in 1 s (FEV_1_) at 58% of the predicted value and a forced vital capacity (FVC) at 53% of the predicted value, with an FEV_1_/FVC ratio of 71% (Figure 1). These findings are consistent with a diagnosis of GOLD grade 2 chronic obstructive pulmonary disease (COPD) and suggest a non-exacerbator phenotype. Consequently, the patient was prescribed an inhaler containing a long-acting β-agonist and a long-acting muscarinic antagonist (LABA/LAMA).

**Figure 2:**
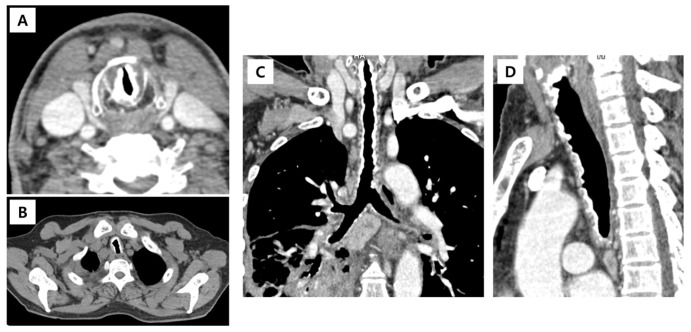
Computed tomography (CT) images of the airway in a patient with tracheobronchopathia osteochondroplastica. (**A**) Axial CT image showing marked calcification at the level of the vocal cords; (**B**) axial CT image demonstrating calcification of the upper trachea; (**C**) coronal CT reconstruction revealing diffuse osteocartilaginous calcification throughout the trachea and main bronchi; (**D**) sagittal CT reconstruction showing diffuse osteocartilaginous calcification involving the trachea and bronchi.

The patient presented with a two-week history of cough, purulent sputum, and fever. Upon examination, bilateral wheezing and rhonchi were observed. Chest computed tomography (CT) revealed extensive calcification extending from the vocal cords to both main bronchi, sparing the posterior wall (Figure 2). Owing to severe airway narrowing, bronchoscopy was performed using thin (4 mm) and ultrathin (3 mm) bronchoscopes.

**Figure 3:**
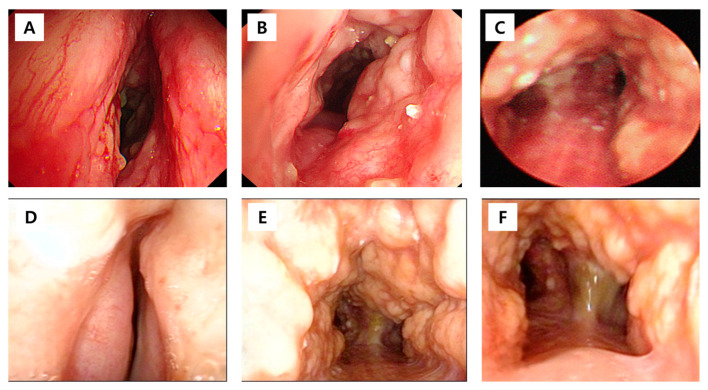
Bronchoscopic findings before and after tracheostomy in a patient with tracheobronchopathia osteochondroplastica. (**A**) Initial bronchoscopy showing vocal cord involvement with mucosal erythema and narrowing; (**B**) initial tracheal view demonstrating diffuse nodular calcified lesions; (**C**) initial carinal view showing irregular mucosal thickening and narrowing; (**D**) follow-up bronchoscopy after tracheostomy showing the vocal cords with a markedly narrowed slit-like opening; (**E**) post-tracheostomy tracheal view demonstrating more prominent nodular calcified lesions; (**F**) post-tracheostomy carinal view showing severe narrowing with persistent calcification.

The procedure revealed diffuse mucosal erythema, spontaneous bleeding, mucopurulent secretions, and necrotic lesions (Figure 3). Cultures from bronchial washings identified *Pseudomonas aeruginosa*, whereas tests for acid-fast bacilli smear, culture, and TB PCR returned negative results. Biopsy specimens indicated chronic inflammatory cell infiltration accompanied by squamous metaplasia. The patient was treated with intravenous antibiotics, leading to resolution of pneumonia and clinical improvement, and was subsequently discharged. Five months later, the patient experienced acute respiratory distress and arrived at the emergency department. Due to severe airway narrowing, endotracheal intubation was not possible.

**Figure 4:**
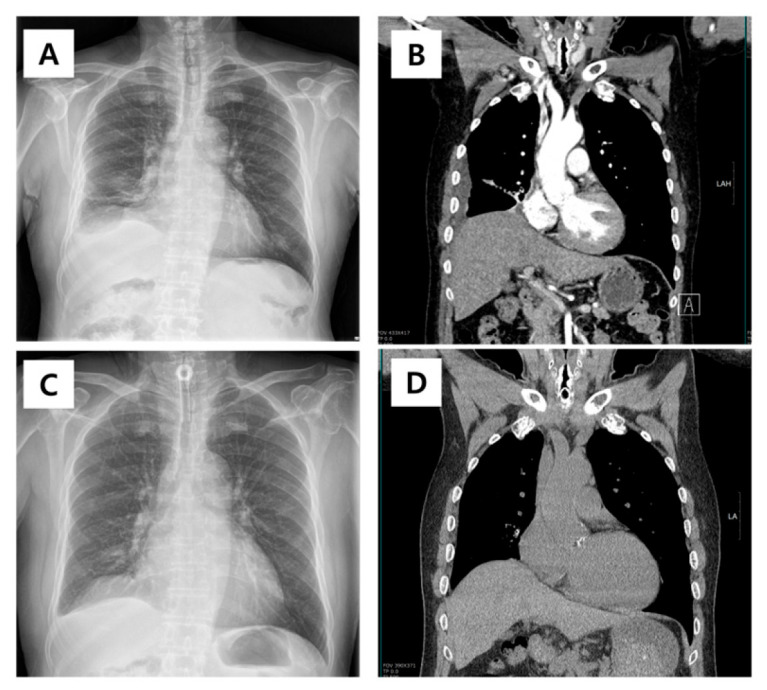
Radiographic and computed tomography (CT) findings before and after tracheostomy in a patient with tracheobronchopathia osteochondroplastica. (**A**) Initial chest radiograph showing partial atelectasis in right lower lobe; (**B**) initial chest CT scan demonstrating airway narrowing and tracheobronchial calcification, predominantly involving the upper trachea and extending to the level of the vocal cords; (**C**) chest radiograph after emergency tracheostomy, with tracheostomy tube in situ; (**D**) follow-up CT scan after tracheostomy.

Consequently, an emergency tracheostomy was performed, and the patient was admitted to the intensive care unit. After stabilization, the patient was discharged with a low-pressure cuffed tracheostomy tube that was subsequently replaced with a cuffless fenestrated tube (Figure 4). The patient has made a full recovery from pneumonia and is currently under outpatient observation, maintaining a stable condition without any notable deterioration in symptoms. While the option of tracheostomy closure was evaluated, the patient opted to retain the tracheostomy, and thus, the tracheostomy tube remained in place.

**Figure 5:**
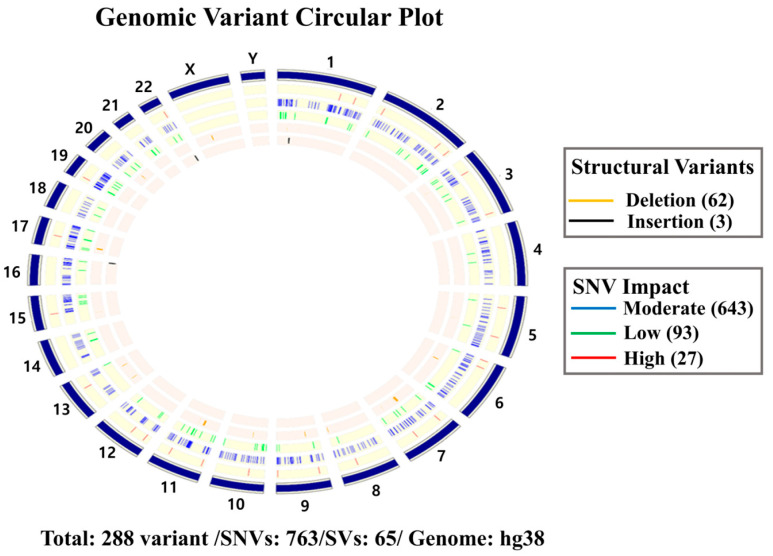
A circular plot illustrates the chromosomal distribution of germline single-nucleotide variants (SNVs) and structural variants (SVs) detected in peripheral blood WGS of the patient. The outermost track represents human chromosomes (hg38). The second track displays structural variants, with orange indicating deletions and black indicating insertions. The third track shows SNVs categorized by predicted functional impact: green for moderate (*n* = 643), blue for low (*n* = 83), and red for high impact (*n* = 27) variants. In total, 828 variants were identified, including 763 SNVs and 65 SVs. High-impact variants were detected in genes involved in coagulation, inflammation, calcium homeostasis, and mucosal barrier function (e.g., *KNG1, PLA2G4D*, *TMEM67*, *CDKN2B-AS1*, *MYLK4*), along with structural variations affecting *TRPV2*, *SPRY2*, *AHNAK2*, *BAZ1B*, and *MUC12/MUC19*. These genomic alterations may contribute to a local immune–stromal microenvironment that promotes dystrophic calcification in the tracheobronchial wall.

To explore the genetic underpinnings of TO, whole blood was collected from the patient and analyzed using whole-genome sequencing (WGS), which was conducted for the first time in the context of TO. The analysis revealed 766 germline mutations, of which 27 were classified as high-impact variants, including nonsense, frameshift, and splice-site mutations (Figure 5). Notable candidate genes included *KNG1*, involved in coagulation and inflammation pathways; *PLA2G4D*, regulating arachidonic acid metabolism and extracellular matrix remodeling; *TMEM67*, a ciliary protein critical for Wnt/Hedgehog signaling and mineralization processes; *CDKN2B-AS1*, an lncRNA strongly linked to vascular calcification and atherosclerosis; and *MYLK4*, implicated in smooth muscle contraction and vascular tone regulation (Table 1).

Additionally, 66 patient-specific structural variations were detected, including gene fusions, deletions, and stop-gain mutations. Candidate genes included *TRPV2*, a calcium-permeable channel linked to osteogenic differentiation; *SPRY2*, a modulator of RTK-MAPK/ERK signaling whose disruption may promote ectopic calcification; *AHNAK2*, a regulator of TGF-β/SMAD signaling associated with fibrosis and calcification; *BAZ1B*, associated with hypercalcemia in Williams–Beuren syndrome; and *MUC12*/*MUC19*, mucin genes whose defects may impair epithelial barrier function and predispose to chronic inflammation and airway calcification (Table 2).

## 3. Discussion

Tracheobronchopathia osteochondroplastica has long been considered a slow-progressing and indolent condition, with most cases being incidentally discovered during bronchoscopy or imaging conducted for unrelated reasons. Nonetheless, growing evidence indicates that TPO represents a diverse clinical spectrum, ranging from asymptomatic incidental findings to severe airway compromise.

This case illustrates an exceptionally rare occurrence of TO, which extends from the vocal cords to the bronchi, leading to pneumonia and acute airway obstruction that required an emergency tracheostomy. Although TO is often seen as a benign and slowly advancing condition, this report highlights that when the disease spreads to the upper airway, particularly affecting the vocal cords, it can result in a life-threatening airway obstruction (Table 3). This underscores the critical need for vigilant long-term monitoring and timely airway intervention in patients with significant TO involvement.

Acute respiratory failure, though exceptionally rare, is a clinically significant complication associated with TO. To date, there have been only a few documented instances where TO has directly led to life-threatening respiratory failure, necessitating intensive airway management, as detailed in Table 3. These reports include cases of acute hypercapnic respiratory failure due to superimposed subglottic abscess formation [6], respiratory insufficiency associated with diffuse tracheobronchial involvement and pneumonia [21], and severe fixed tracheal stenosis resulting in failed intubation and emergency tracheostomy [20]. Although the precipitating factors differ, these cases share significant clinical features with our patient, such as extensive airway involvement, inflammatory changes observed in histopathology, and acute decompensation triggered by infection or airway compromise. Together, these observations indicate that acute respiratory failure in TO is not simply a result of static structural narrowing; rather, it reflects the interaction between chronic airway remodeling and superimposed inflammatory insults.

The second and most academically significant aspect of this case is that, to the best of our knowledge, this is the first instance in the literature where WGS has been applied to TO. Previous research on TO has primarily concentrated on describing clinical features, radiologic and bronchoscopic findings, and histopathological characteristics, with some hypotheses suggesting roles in chronic inflammation and metabolic disturbances. However, no genomic investigations were conducted prior to this case. Through WGS, we identified germline mutations in *KNG1*, *PLA2G4D*, *TMEM67*, *CDKN2B-AS1*, and *MYLK4*, as well as structural variations in *TRPV2*, *SPRY2*, *AHNAK2*, *BAZ1B*, and *MUC12*/*MUC19*. These findings implicate the pathways associated with inflammation, coagulation, calcium homeostasis, ciliary function, vascular smooth muscle tone, and mucosal barrier integrity.

An especially significant observation is that, despite the presence of systemic germline mutations, calcification was restricted to the airway. This strongly indicates that germline alterations alone are insufficient to drive the disease phenotype. Instead, local microenvironmental factors seem to play a crucial role. The histopathological examination of TO tissues uncovered the presence of inflammatory cells, notably lymphocytes and macrophages. These cells are recognized for their role in releasing cytokines and reactive oxygen species, which facilitate tissue damage and remodeling. Simultaneously, fibroblasts within the submucosa are activated, contributing to the remodeling of the extracellular matrix and establishing a favorable environment for calcium deposition. These observations support a model in which systemic genetic predisposition collaborates with local immune and stromal cell activity to drive dystrophic calcification localized to the airway.

**Figure 6:**
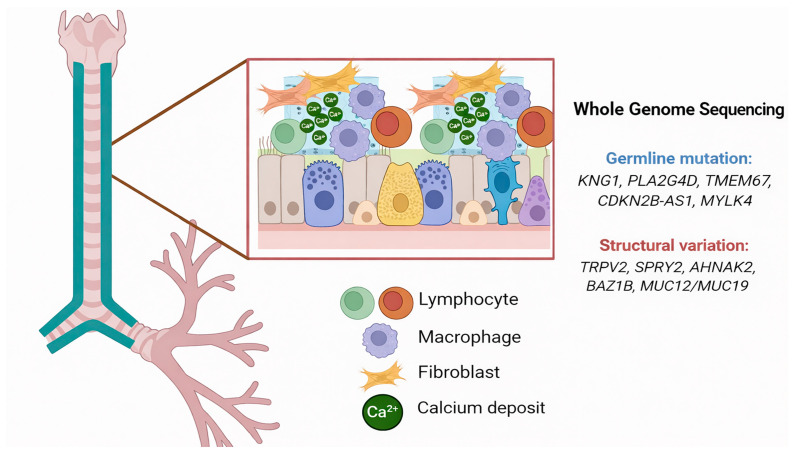
Proposed schematic illustration of airway calcification in a patient with tracheobronchopathia osteochondroplastica. Whole-genome sequencing (WGS) identified germline mutations (*KNG1*, *PLA2G4D*, *TMEM67*, *CDKN2B-AS1*, and *MYLK4*) and structural variations (*TRPV2*, *SPRY2*, *AHNAK2*, *BAZ1B*, *MUC12*/*MUC19*), which are implicated in coagulation, inflammation, calcium homeostasis, extracellular matrix remodeling, and mucosal barrier function. At the tissue level, lymphocytes and macrophages infiltrate the submucosa, while fibroblasts contribute to extracellular matrix remodeling. Together with genetic alterations, these immune and stromal cells may create a microenvironment that favor calcium (Ca^2+^) deposition, ultimately leading to dystrophic calcification restricted to the airway.

From a translational perspective, this case underscores the significance of integrating multi-omics approaches to further elucidate the molecular underpinnings of TO. Future studies utilizing transcriptomic, epigenomic, and proteomic profiling could reveal how systemic germline mutations interact with tissue-specific factors to induce airway-restricted calcification. Such investigations may not only provide novel insights into TO pathogenesis but also shed light on other disorders characterized by ectopic calcification (Figure 6).

### Literature Review

The precise prevalence of TO remains elusive, primarily due to its often asymptomatic presentation and incidental identification. Prevalence rates, as estimated by autopsy and bronchoscopic studies, range from 0.05% to 0.3% [22]. The disease is most frequently diagnosed in middle-aged to elderly individuals, with several cohorts reporting a slight male predominance [20,23]. Although many patients are asymptomatic, symptomatic cases typically present with chronic cough, exertional dyspnea, wheezing, recurrent lower respiratory tract infections, hemoptysis, or voice changes [4,23,24].

Upper airway involvement, particularly when it extends to the subglottic region or vocal cords, is exceptionally rare. Most cases documented in the literature are restricted to the anterior and lateral walls of the trachea and proximal bronchi, with the posterior membranous wall typically remaining unaffected [23,25]. Vocal cord involvement has been documented only in isolated case reports, yet it holds clinical significance due to the heightened risk of critical airway obstruction and the increased procedural challenges during endotracheal intubation [26,27].

CT is an essential diagnostic tool, often revealing irregular nodular thickening of the tracheobronchial wall, accompanied by calcified or ossified protrusions extending into the airway lumen [28,29]. Bronchoscopy reveals the classic “cobblestone” or “rock-garden” appearance caused by firm, whitish nodules beneath an intact or inflamed mucosa [30,31]. These nodules are frequently friable and susceptible to bleeding, which may complicate diagnostic biopsy or therapeutic interventions. Histopathologically, TO is characterized by submucosal cartilage and mature bone formation, often accompanied by chronic inflammatory infiltrates, squamous metaplasia, and varying degrees of fibrosis [20,24].

From a therapeutic standpoint, the management of TO remains primarily supportive, as no treatment has been established to modify the disease [20,23]. Medical treatment primarily targets secondary complications, such as recurrent infections or coexisting obstructive airway disease, while pharmacological interventions do not seem to affect the underlying osteochondral remodeling [23,30]. In cases of focal obstruction, bronchoscopic interventions may serve as a means to achieve relief from symptoms [30,32]. Significantly, the presence of severe airway rigidity or involvement of the upper airway may render endotracheal intubation unfeasible, necessitating an emergency tracheostomy, as demonstrated in the current case and documented in previous reports [6,33]. These findings highlight the critical need for early identification of TO in patients at risk of airway compromise, along with proactive planning for airway management.

The pathogenesis of tracheobronchopathia osteochondroplastica is primarily characterized by chronic inflammation of the airways and the metaplastic transformation of submucosal tissues into osteocartilaginous nodules. Notably, there is no evidence of specific germline mutations or polymorphisms associated with this condition [22,25,34,35,36]. Clinical series involving both adults and children highlight recurrent respiratory infections, exposure to irritants, and smoking as triggers. Biopsies from these cases reveal inflammatory infiltrates that progress to elastic fibrosis, cartilage formation, and ossification, following the models of Virchow’s ecchondrosis or Aschoff’s elastic metaplasia [22,35].

Immunohistochemical investigations reveal that the upregulation of Bone Morphogenetic Protein-2 (BMP-2) and Transforming Growth Factor (TGF)-β1 plays a crucial role in mediating ectopic osteogenesis. The study by Tajima et al. illustrates that the interaction between these proteins is instrumental in the development of calcific foci in TO lesions [37]. Recent research by Hong et al. highlights the dysfunction of tracheobronchial basal stem cells, revealing their role in mediating ectopic bone and cartilage formation under inflammatory conditions. The inflammatory characteristics observed in these basal stem cells underscore the pivotal role of chronic inflammation in driving mesenchymal shifts toward chondrogenic and osteogenic differentiation [38].

The prognosis for airway stenosis depends on the condition’s severity, with non-progressive cases being the most common in long-term pediatric studies [39,40]. Nonetheless, there has been a reported case of a 6-month-old male infant who required interventional treatment due to severe stenosis of the upper trachea [41]. Genetic factors remain unproven across cohorts, framing TO as primarily an inflammatory-degenerative process modulated by airway basal stem cell malfunction and BMP-2/TGF-β signaling [25,37,38].

While TO is traditionally considered non-progressive, there are increasing reports of cases with significant airway involvement, resulting in persistent tracheal stenosis, repeated pneumonia, and acute respiratory failure [5,6,20,21]. Acute decompensation is frequently triggered by superimposed infection, mucosal edema, or hemorrhage, underscoring the dynamic interplay between static structural narrowing and inflammatory insults. Several case reports highlight instances of failed endotracheal intubation and the subsequent necessity for emergency tracheostomy due to pronounced tracheal rigidity and luminal narrowing [26,27,42]. These cases emphasize the critical need to consider TO as a potential factor in challenging airway management, especially in elderly patients with unexplained airway obstruction.

To date, the literature on TO has been almost exclusively descriptive, focusing on clinical, radiologic, and histopathological features. Systematic investigations into genetic susceptibility or molecular drivers have been notably absent. This absence of genomic data represents a major gap in the existing body of knowledge. Given the striking osteochondral phenotype of TO, parallels have been drawn with disorders of ectopic calcification and abnormal bone formation; however, such comparisons have remained speculative in the absence of molecular evidence.

In this context, the current case significantly contributes to the literature by identifying high-impact germline variants and structural variations in genes associated with inflammation, coagulation, calcium homeostasis, ciliary signaling, smooth muscle function, and epithelial barrier integrity. This case introduces a novel genetic perspective to TO research. Notably, the restriction of calcification to the airway, despite systemic germline variants, supports a multifactorial disease model. In this model, genetic predisposition interacts with local microenvironmental factors—such as chronic inflammation and stromal remodeling—to drive tissue-specific ectopic ossification.

## 4. Conclusions

We present a novel and unprecedented case of TO extending to the vocal cords, complicated by pneumonia and acute respiratory failure necessitating emergency tracheostomy. In addition to its clinical rarity, this case is notable for being the first to employ WGS analysis in TO, thereby paving the way for new insights into the molecular basis of TO.

WGS results revealed germline mutations and structural variants in genes linked to coagulation, inflammation, calcium homeostasis, extracellular matrix remodeling, and mucosal barrier function. These findings suggest that genetic predisposition may contribute to abnormal calcification processes. However, the restriction of calcification to the airway, despite the presence of systemic germline mutations, underscores the critical role of local microenvironmental factors, including immune cell infiltration and fibroblast-driven remodeling.

This integrated clinical, histological, and genomic analysis redefines TO not merely as a rare benign airway disorder, but as a complex disease involving both systemic genetic susceptibility and tissue-specific pathogenic mechanisms. Moving forward, accumulating multi-omics data and expanding genomic research on TO is essential for clarifying its pathogenesis. Ultimately, such insights may enable the development of precision medicine strategies and guide both early diagnosis and targeted interventions for this challenging condition.

## Figures and Tables

**Table 1:** High-impact Germline Mutations Identified in the Patient.

Gene	Variant Type	Functional Effect	Pathway Involved	Clinical Implication	Ref.
*KNG1*	Nonsense	Coagulation/inflammation dysregulation	Tissue damage, vascular permeability	Dystrophic calcification	[8]
*PLA2G4D*	Missense	Altered arachidonic acid metabolism	Chronic inflammation, ECM remodeling	Tissue calcification	[9]
*TMEM67*	Splice site	Ciliary dysfunction	Wnt/Hedgehog signaling	Abnormal mineralization	[10,11]
*CDKN2B-AS1*	Frameshift	Dysregulated lncRNA function	Cell proliferation, atherosclerosis	Vascular calcification	[12]
*MYLK4*	Missense	Smooth muscle dysfunction	Vascular tone regulation	Coronary artery calcification	[13,14]

**Table 2:** Significant Structural Variations Identified in the Patient.

Gene	SV Type	Functional Effect	Pathway Involved	Clinical Implication	Ref.
*TRPV2*	Gene fusion	Calcium channel dysfunction	Osteogenic differentiation	Ectopic calcification	[15]
*SPRY2*	Deletion	ERK/Wnt signaling alteration	Osteogenesis modulation	Promotion of abnormal calcification	[16]
*AHNAK2*	Insertion	Dysregulated TGF-β/SMAD signaling	Fibrosis, inflammation	Calcification-prone microenvironment	[17]
*BAZ1B*	Deletion	Calcium homeostasis defect	Hypercalcemia	Soft tissue calcification	[18]
*MUC12/MUC19*	Gene fusion	Impaired mucosal barrier	Chronic epithelial injury	Tracheobronchial calcification	[19]

**Table 3:** Reported Cases of Tracheobronchopathia Osteochondroplastica Presenting with Acute Respiratory Failure.

First Author (Year)	Patient Characteristics	Finding	Management	Outcome	Ref.
Leske (2001)	41-patient cohort; one highlighted case	One patient presented with acute respiratory failure; intubation attempts failed due to severe tracheal stenosis	Emergency tracheostomy	Maintained on long-term home mechanical ventilation because of severe tracheal stenosis	[20]
Hantous-Zannad (2003)	42-year-old male, non-smoker	Tracheal narrowing with consolidation of LLL; extensive and multiple grey/white nodules with scattered calcifications in the main and lobar bronchi	Oxygen therapy; antibiotics	Improved; resolution of consolidation	[21]
Danckers (2015)	27-year-old male	Acute hypercapnic respiratory failure; subglottic mass with cystic and solid component obstructing 75% of the airway	Intubation, mechanical ventilation; surgical drainage; prophylactic tracheostomy; antibiotics	Asymptomatic after tracheostomy removal; persistent vocal cord fixation	[6]

## Data Availability

The data presented in this study are available on request from the corresponding author. The data are not publicly available due to privacy and ethical reasons.

## Abbreviations

The following abbreviations are used in this manuscript:

TO	Tracheobronchopathia osteochondroplastica
WGS	Whole-genome sequencing
mMRC	Modified Medical Research Council Dyspnea Scale
GOLD	The Global Initiative for Chronic Obstructive Lung Disease
SNVs	Single-nucleotide variants
Indesls	Insertions/deletions
VEP	Variant Effect Predictor
SVs	Structural variants
